# Narrative Insights and Perspectives Into Burnout and Wellness Among U.S. Orthopedic Surgery Residents: The Influence of Gender and Minority Identification

**DOI:** 10.7759/cureus.109635

**Published:** 2026-05-25

**Authors:** Michael Hall, Peyton G Sakelaris, Kelechi Nwachuku, Ikenna Nwachuku, Sukanta Maitra, Jenifer Powers, Cameron Harris, Kian Habashi, Angelina Vera

**Affiliations:** 1 Orthopaedic Surgery, Kirk Kerkorian School of Medicine at University of Nevada, Las Vegas, Las Vegas, USA; 2 Orthopaedic Surgery, University of California, San Francisco, San Francisco, USA; 3 Orthopaedic Surgery, New York University Langone Medical Center, Veterans Affairs New York Harbor Healthcare System, New York, USA; 4 Orthopaedic Surgery, University of New Mexico School of Medicine, Albuquerque, USA

**Keywords:** burnout, diversity and inclusion, orthopedic surgery residents, resident well being, underrepresented minority residents

## Abstract

Background

Completing an orthopedic surgery residency accredited by the Accreditation Council for Graduate Medical Education requires managing academic, clinical, and personal duties. For underrepresented minorities (URMs), additional challenges related to ethnicity, gender, religious affiliation, and socioeconomic status contribute to burnout. Despite initiatives to address well-being, burnout rates remain high. This qualitative study uses a narrative approach to explore how URM orthopedic residents experience burnout, with a focus on psychosocial stressors.

Methodology

We conducted semi-structured interviews with 20 URM orthopedic residents using the Consolidated Criteria for Reporting Qualitative Research framework for qualitative research. Data were analyzed thematically, identifying six key themes.

Results

Findings include the impact of intersectional fatigue, magnifying glass phenomena, self-fulfilling prophecy, and stereotype threat. Participants highlighted the emotional toll of code-switching and feelings of isolation.

Conclusions

Suggested interventions included promoting diversity in leadership, fostering supportive peer relationships, and organizing wellness events. This study emphasizes the need for inclusive programs that reduce burnout, enhance well-being, and support the retention of URMs in orthopedic surgery.

## Introduction

Completing an Accreditation Council for Graduate Medical Education (ACGME)-accredited orthopedic surgery residency requires managing educational curricula, patient care, surgical duties, medical documentation, and research participation. For underrepresented minorities (URMs), a “hidden curriculum” may also be necessary for success in orthopedic training [1]. According to a 2023 study, the Hispanic/Latino, African American, Native American, Multiracial, and female populations are underrepresented in orthopedic surgery when compared to their proportion in the U.S. population [2]. Life outside of work adds further complexity, requiring residents to balance education, duties, and personal life.

In 2017, the ACGME mandated that residency programs focus on resident well-being, providing strategies to help cope with emotional exhaustion and burnout [3]. Despite work hour restrictions, burnout rates remain high, at approximately 40-56% [4]. Defined as a prolonged response to emotional and interpersonal stressors, burnout involves exhaustion, cynicism, and inefficacy [4]. It contributes to mental fatigue, feelings of incompetence, and depersonalization, which can lead to medical errors and poor performance on the Orthopedic In-Training Examination [4]. Addressing resident wellness can be challenging due to its subjective nature and varying preferences based on individual hobbies, beliefs, and interests [5].

Studies on the relationship between URM status and burnout show mixed results. Some studies suggest URMs experience higher rates of burnout than non-URMs, while others report no difference [6-14]. These studies often involve cross-sectional data from non-medical university faculty, medical students, and residents across various specialties, which may not reflect the unique challenges faced by surgical residents [6-14]. More research is needed on burnout in URM surgical residents, particularly through qualitative studies.

Studies consistently highlight psychosocial barriers faced by URM orthopedic surgeons [15-20]. URMs, especially Black individuals and women, have the highest attrition rates in orthopedics and other surgical specialties (12% and 7.5%, respectively) [21]. Contributing factors include lack of support, exclusion, stress, burnout, and deteriorating clinical performance [22]. Despite making up 35% of the general population, URMs represent only 11% of orthopedic residents and 7% of practicing attendings [21]. Similarly, while women comprise 50% of the population, they make up only 18% of orthopedic residents and 5.9% of practicing orthopedic surgeons [22]. Research shows that male and female applicants to orthopedic residency programs exhibit comparable academic qualifications, and URM candidates are similarly competitive [23]. However, race, rather than gender, was found to be independently associated with lower odds of admission [24-26]. These findings suggest that the higher attrition rates among URM residents may stem from non-academic factors, such as burnout.

Orthopedic surgery residency involves more than clinical knowledge; it requires interpersonal skills to navigate diverse backgrounds, cultures, and beliefs [5]. To our knowledge, no study has examined the intersection of ethnicity, gender, minority status, and religious affiliation on residency burnout using a first-person narrative approach.

This qualitative study aimed to explore how gender, race/ethnicity, religion, and other marginalized identities shape burnout and wellness among orthopedic surgery residents. It also attempts to identify recurring themes that may inform program-level interventions.

## Materials and methods

Participants and research team

To standardize this qualitative study, the Consolidated Criteria for Reporting Qualitative Research (COREQ) framework was utilized [27]. Selective sampling was used to recruit participants who identify as URM in orthopedic surgery. We defined our population to include residents who are underrepresented in orthopedic surgery, residents with minority identities, and residents with minority religious beliefs in the U.S. These populations include Black, Hispanic, Native American, Asian, and female residents. We also included residents with minority identities, including Asian residents and Caucasian residents with non-Christian religious beliefs. Voluntary sampling was performed through Nth Dimension and Gladden Society, supplemented by convenience sampling via social media and text messaging. Snowball sampling was also employed, where participants were asked to refer additional candidates. Recruitment material was framed to welcome a broad range of resident experiences, regardless of burnout status or DEI initiative involvement. A total of 20 residents from eight U.S. orthopedic surgery programs participated, providing verbal consent before interviews. Interviews took place from July to December 2024.

Study design

Video interviews were conducted via Zoom and recorded for no longer than 40 minutes, with no repeats. Otter transcription software was used strictly to transcribe the Zoom interviews verbatim. To enhance validity and reduce bias, a diverse team of interviewers was used: one resident (M.H., Black male) and two medical students (K.H., Asian male, and C.H., White female). The identity of the medical position the interviewer was in was not revealed to the interviewees. Initial demographic data was collected, including religious affiliation, ethnicity, gender, year of training, location, and socioeconomic background. The semi-structured interviews then explored themes such as burnout and the impact of minority status on orthopedic training, with the interview questions listed in the Appendices. The study was approved by the University Medical Center Institutional Review Board.

Data analysis

Thematic analysis was used, applying constant comparative methods to identify recurring themes in the transcripts [28]. An initial codebook was developed after an open-coding session of three transcripts, and the codebook was iteratively refined with the rest of the transcripts. Data analysis was conducted by two reviewers (K.H. and C.H.), and disagreements were resolved by a third reviewer (M.H.). Transcripts were not returned to participants for review.

## Results

**Figure 1:**
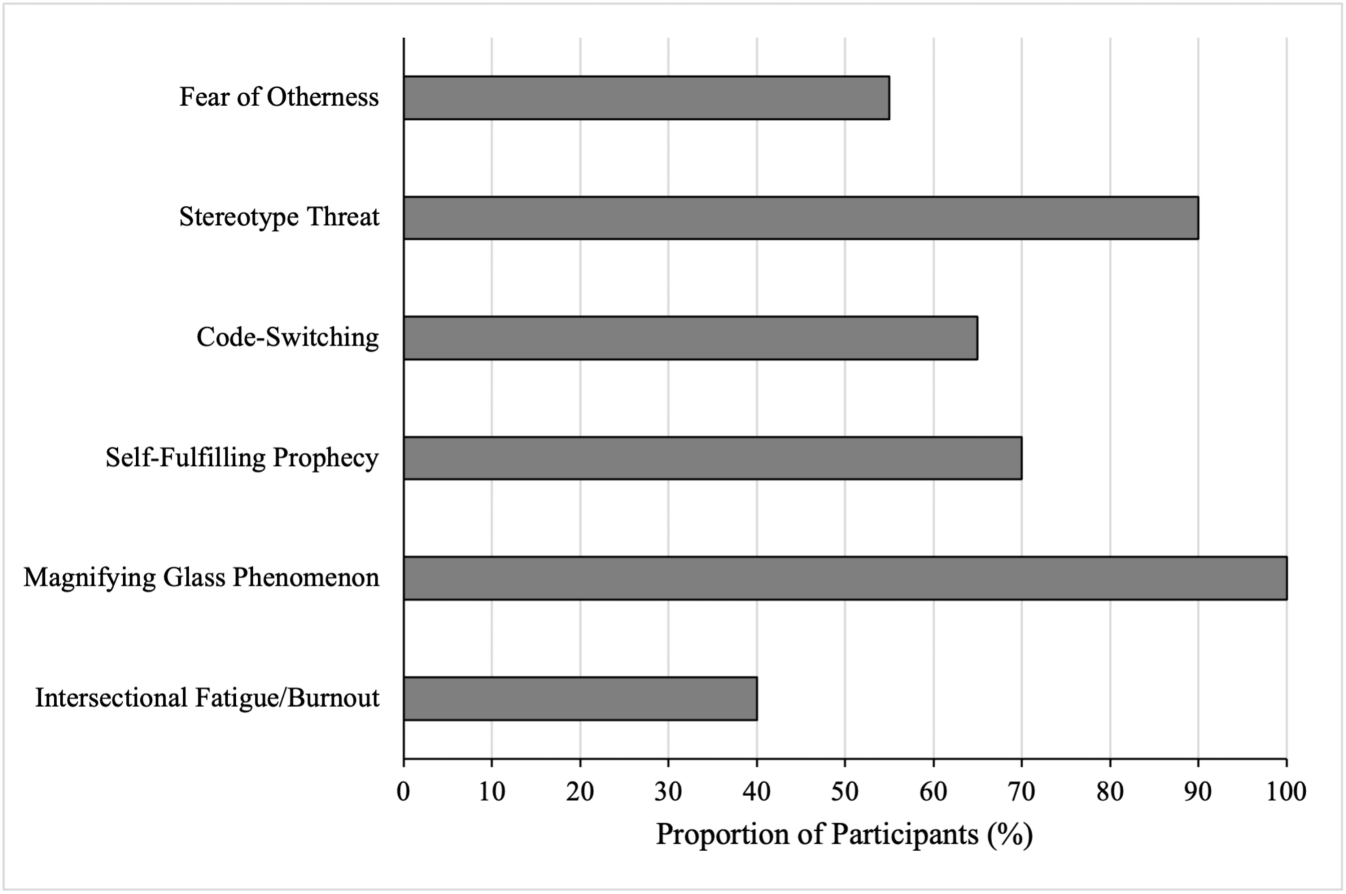
Proportion of participants reported in % who experienced each of the six themes: intersectional fatigue/burnout, magnifying glass phenomenon, self-fulfilling prophecy, code-switching, stereotype threat, and fear of otherness.

A total of 35 individuals were invited or expressed initial interest. Of these, 15 declined participation or did not respond to follow-up. This resulted in 20 participants who consented and were interviewed for this study. The demographic, ethnic, and religious affiliation information of the orthopedic surgery residents is presented in Table 1. Data saturation was reached at interview number 17 as no new themes were introduced following this interview. Six key themes were identified upon analysis: intersectional fatigue/burnout, magnifying glass phenomenon, self-fulfilling prophecy, code-switching, stereotype threat, and fear of otherness. A description of each theme is detailed in Table 2. The most common theme found among participants was the magnifying glass effect, with 100% of participants reporting this experience, while the least common theme found among interviewees was intersectional fatigue/burnout, with 40% of participants reporting this experience. The proportion of participants who reported experiencing each theme is depicted in Figure 1. All participants were orthopedic surgery residents at ACGME-accredited programs in the U.S. Direct quotes and excerpts from residents, aligned with the themes identified, are provided in Table 3. The themes and corresponding quotes are discussed below, along with interventions suggested by interviewees.

**Table 1:** Study participants’ demographics.

Demographic variable	Number of participants
Gender	
Male	13
Female	7
Ethnicity/Race	
African American	8
Asian	4
Hispanic	4
Caucasian	4
Religious affiliation	
Non-denominational Christianity	7
Agnostic	1
Roman Catholic	1
Church of Jesus Christ of Latter-Day Saints	3
Seventh-Day Adventist	2
Buddhism	2
Hinduism	2
Islam	1
Judaism	1

**Table 2:** Qualitative themes and definitions acquired from underrepresented minority-interview responses. References: [29-35].

Qualitative theme	Theme definition
Intersectional fatigue/burnout	The concept of intersectional or “racial” fatigue is characterized by the emotional and mental exhaustion experienced by individuals due to discrimination and racial or gender stressors from repeated micro and macro-aggressions
Magnifying glass phenomena	The “magnifying glass” concept implies how behaviors and mistakes created by particular individuals are disproportionally scrutinized and magnified due to their status or identity
The self-fulfilling prophecy	The “self-fulfilling prophecy” phenomenon occurs when another person’s expectations lead to behaviors that result in expectations coming to fruition, even if initial expectations or biases are incorrect or unjust
Code-switching	“Code Switching” is a linguistic and social phenomenon that occurs when an individual alternates between styles of communication or dialect depending on the context and interaction
Stereotype threat	“Stereotype threat” occurs when individuals who are aware of negative stereotypes experience anxiety in fear of confirming such stereotypes or affirmations
Fear of otherness	The “fear of otherness” occurs when anxiety and discomfort arise within individuals or groups that are perceived as different from themselves, particularly in terms of culture, religion, ethnicity, or values

**Table 3:** Themes gathered from the underrepresented minority-resident interviews with their substantiating quotes.

Qualitative theme	Direct participant quote
Intersectional fatigue/burnout	“Is the reason that I’m not getting autonomy in the operating room or having positive interactions with faculty because I’m a bad surgeon or is it because I’m black and a woman? That is on my mind every day, so when I come home and, pre op plan, I’m still thinking of that. I can’t put that aside and think just pure orthopedics and retain information.” “When studying for OITE, my mind is constantly bombarded by these other factors that I feel are out of my control and I let others negative biases or perceived stereotypes of me affect my performance. I wonder how much extra brain space really gets taken up by, all other “non-orthopedic stuff.” I feel a mental fog daily. After nine hours of sleep, and I’m still tired because I’m still thinking about, like, I said, those non orthopedic stressors that minorities and females face on a daily basis.” “What would our learning capacity be if we did not have to worry and stress about those non ortho things? And just like from an energy standpoint, or from a brain space standpoint, when you compound that with life outside the hospital, right? Finances, if you have a family, you know, you live on your own if family’s not around, and, you know, religious aspect, and how you cope with, like, it’s, it’s, it’s a lot, you’re right, the mental capacity over five-year span, just for our own wellness, right?”
Magnifying glass phenomena	“I make mistakes, which everybody does but then because of the positive or negative or mostly negative reputation, then, you have this microscope or magnifying glass on you. So I feel like we start out with a magnifying glass on us with a late start already and everybody is waiting for you to make a mistake; so they capitalize on an opportunity to penalize you on it and call you out on it during your evaluations.” “I’ve seen this happen; people are so fast to give you a reputation. I think especially as a person of color, or as a woman in the program. For example, there is a white woman in my class and they’re so quick to call her ‘annoying and bossy’ and all these things and I’m thinking is she my favorite person: maybe not but she objectively, is doing the same thing that other people men or women are exactly doing.” “To me, if a program really is about diversity and inclusion; then it will show in their faculty because that is something from a recruiting and retention standpoint they can control; residents yes but to lesser extent because they can rank the URM or female resident #1 but that same resident may not rank the program #1. So, I feel we are even scrutinized and under a microscope when we get here because we are ‘rare’ for lack of better of terms.”
The self-fulfilling prophecy	“I think there’s a lot of implicit bias against URMs and an assumption that they are lesser than or not worthy of their position. And so that becomes a self-fulfilling prophecy. When you think that people think you’re not as good, then you start to think that and you do not perform as good, or you have convinced yourself you're not performing as well, even though you are.” “Early on, those residents with a ‘good reputation’ have a ‘longer leash,’ they have been offered a lot more opportunities to operate, a lot more autonomy, a lot more opportunities to make mistakes then some of the rest of us. While those with a ‘bad reputation’ are given a ‘short leash.’” And then as soon as those things (mistakes) happen to me, whether it’s making a mistake or being hesitant and because you’re just not sure, they immediately think that you just have no idea what you’re doing and they (attendings) take it away from you. So then when you don’t have a chance to build surgical skills over time, your imposter syndrome worsens.” “My mom is from Honduras, my dad is Portuguese, so I have both Portuguese and Spanish heritage. My mom works at a grocery store. I help support her in many ways; my parents aren’t physicians like most of my co-residents. Coming to residency, I had this, how should I say, impostor syndrome? I think we all face that, whether it’s underrepresented minorities are not, you know, like, oh, should I be here? I always had a chip on my shoulder, like, I always I grew up with, you know, a single mom, basically, I grew up in the projects first in my family to even go to college. If I’m coming from the streets, like coming from the projects, and getting to this point in life, then, you can do that. It’s cliche, but it’s true, the American dream, you know, you come from, like, wherever you come from, you know, you plant your seed, especially for us minorities, it’s hard to be the first to establish that generational wealth or success without any guidance before you. It took me a while to get to this point in life. For example, I’m not the best test taker, multiple attempts to get accepted to medical school and into residency, I wasn’t the epitome, or the echelon of like, you know, a step one in the 280s, I’m not bad but what's gotten me to this point in life is my grit and how much work and sacrifice I put into where I am. I try to fight against self-fulfilling prophecies all the time because I know if give into them mentally then my performance will suffer and I do not want to let down myself, my mother and all my culture and my people who I hope to trailblaze for.”
The code-switching tactic	“I’m black but grew up lower middle class, played soccer growing up, lived in suburbia; went to school in pre-dominantly white areas but I had a diverse group of friends, white, Hispanic, black Asian, etc.; so I’ve learned to ‘code switch’ interacting with particular groups of people both in school, sports, work and now residency. I feel those URM residents who can ‘code switch’ and adapt to interacting with people of all backgrounds are able to better cope with the interpersonal/social challenges of residency. But also I feel it sort of disappointing that we cannot be ourselves completely, I know there is a time to be cordial and professional in the workplace in comparison to out of it but I see other residents and attending say something or act some way and I’m like: if I said or did something like that. I would be ousted and judged.” “When I think about it, I have had to ‘code switch’ a ton. Obviously, most of the people in my program do not look like me, talk like me and come from the same culture as me. I am black and a first-generation college student; I change the way I talk when I am around co-residents and faculty in comparison to with my family and friends outside of residency, I must be more ‘proper’ for lack of better terms. I listen to music that is different than what my attendings or co-residents listen to so I feel like I have to ‘water myself down’ around them and stick ‘just to business,’ like when for example, country and soft rock comes on in the OR; like I have no idea what the songs are when they ask me; so I find myself listening to it more than I would just so I could have a chance. It is mentally exhausting having to expend energy daily being someone I am not and try ‘to fit in’ and ‘code switch’; I feel minorities experience greater stress because of this.”
Stereotype threat phenomena	“You don’t want to say, ‘you already kind of got this,’ you know like have this ego. Like, you know, I’m the only black guy, in the class, so you don’t want to make it seem like you’re the one that always needs help, right? Because that’s that stereotype. Like, of course, he needs help. So, you feel like you need to kind of grind and figure it out, right? So that’s probably more so because of my ego, rather than, like other people, making me isolated but I feel this sort of pressure not to ask for help and reinforce the stereotype of the black guy needing help all of time.” “Stigmas with every culture and every background exist, you’re fighting those stigmas while still trying to stay true to yourself. I would use my dad as an example; he is an Indian doctor; I am Indian. He never leaves the house without being cleanly shaven. He never leaves the house without being showered, perfectly groomed, smelling great. And I never understood why. Then I realized, you know, there was a stigma, and there may still be a stigma in parts of the country, about Indian men. He had a response to that, he felt he had a responsibility to both himself and to his culture to fight against it, because he knew he was fighting that every time he walked into a room, he’d be cleanly shaven, he had to be dressed perfectly. Perfect tie, like double Windsor. These are the things that I was taught. And I was like, this makes no sense. I only realized when I got into training that that’s what he was fighting this whole time. I didn’t identify it at the time. But that’s what you must do. You have to figure out what people are going to assume about you, and you need to make sure you crush it. So that they’re one convinced that this is not the norm and they take you seriously and do not generalize you as ‘just another one of them.’”
Fear of otherness	“I’m black and non-denominational Christian; never really interacted with Latter Day Saints (LDS) church growing up. Moving here first thing, I noticed starting is how large the LDS community is here. Interacting with attendings who are of the LDS faith has actually been just fine. Even though they have been nothing short of being amazing and professional and nothing short of cordial; some of their principles and beliefs against people of color has always been in the back of my mind. Matter of fact one of my mentors in my program is LDS and Caucasian and before I came here; I would not have though he would be a mentor of mine but really ‘kicking that barrier’ down and having an open-minded conversation earlier on really helped alleviate that ‘tension’ I was feeling earlier on; and for him to be open minded to it to was very helpful. Now he offers advice, and we can talk about anything, from our faith, beliefs, families, etc. But still unfortunately no matter how connected I feel with this attending; I still can’t remove their views of colored people from my subconscious, and this still creates some anxiety for me personally.” “I identify as Asian and Buddhist, I know there’s a bunch of Mormons in the program, some Catholics and Christians too, but less vocal. I feel like I fit in less compared to the ‘general ortho residents’ in the program. One of the attendings essentially said something along the lines of, like: ‘you know, you’re not a typical ortho resident, you can’t say the same thing that another typical ortho resident can say, it just doesn’t come off the same way.” And I was just like, what? That makes zero sense to me, but there is a difference, and I’m starting to feel it now, not feeling like ‘one of them,’ definitely not something that I noticed when I came in but now it is. I don’t think I’ve built a strong enough relationship with faculty to feel comfortable venting or opening up. Unfortunately, if you have an uncertain bias against you in some way, it just sticks with you and it can be hard, because then they kind of see you in that way and I’ve seen it in our program; if they have a resident who they think is for example, very lackadaisical, that’s just how they treat them, they’re like: oh, he’s, you know, he’s always absent, or doesn’t care, doesn’t participate. They don’t give them a chance or even try to get to know the backstory or life circumstances or experiences/tribulations they are going through on a daily basis.”

Magnifying glass phenomenon

All 20 URM residents felt that their mistakes were often magnified compared to their peers. They described being “under a microscope” from day one of their intern year. This increased scrutiny affected their evaluations and reputation, contributing to heightened performance pressure. Many felt that their errors were unjustly interpreted by faculty or co-residents, which reinforced negative stereotypes. One resident stated, “I’ve seen this happen so often where people are so fast to give you a reputation, especially as a person of color, or as a woman in the program.” Imposter syndrome and heightened scrutiny were believed to contribute to systemic minority and gender disparities in orthopedics, hindering diversity retention.

Stereotype threat

In total, 18 residents discussed experiencing stereotype threat. One resident, who identified as Indian, shared how his father’s meticulous grooming was an effort to combat negative stereotypes about Indian people and hygiene. As a physician, the resident now understands this experience. Female residents also noted stereotype threats regarding physical strength in orthopedic settings. One female resident described how she mentally approaches difficult reductions, stating: “There’s still a stigma with women in orthopedics when it comes to strength. I’m only 5'0” and I get ‘looks’ when I walk into the ER for reductions. I manage to succeed because I’ve learned to utilize technique, but it’s always in the back of my mind that people doubt me because I am not only small but female.”

The self-fulfilling prophecy

Overall, 14 residents shared experiences related to the “self-fulfilling prophecy” from high school and college, which carried into residency. As one first-generation male immigrant resident stated, “Negative stereotypes lead to self-doubt, reinforcing a cycle. We then internalize these stereotypes about our capabilities, which affects our performance and exacerbates imposter syndrome.” Eight residents knew or were co-residents with a URM who had been dismissed from their orthopedic program, with many attributing the self-fulfilling prophecy and imposter syndrome as contributing factors to their difficulties navigating residency.

Code-switching

In total, 13 residents shared experiences in which they participated in code-switching during their residency training. Interestingly, one Black resident described struggles with “code-switching” and how a co-resident, raised in middle-class suburbs, was better able to adapt socially. The co-resident could easily navigate professional and formal environments, whereas the resident from a homogenous, inner-city background found the process more exhausting. Many Black and Latinx residents reported feeling more burnout compared to their non-URM counterparts, as they had to expend more energy creating a “façade” that masked their true identities. One resident stated: “Constantly having to act and monitor one’s presentation, stepping on eggshells, in addition to clinical work, burns someone quick over the span of five years.”

Fear of otherness

Overall, 11 of the residents interviewed experienced a fear of otherness during their orthopedic residency training. Some residents, especially those in geographic areas with a higher-than-average Latter-Day Saints (LDS) population, experienced the “fear of otherness” phenomenon. One non-denominational Christian URM resident described initial interactions with LDS attendings as “awkward and daunting.” He was initially reluctant to discuss non-orthopedic topics for fear of judgment. However, over time, they found common ground, leading to a productive mentor-mentee relationship. To assimilate and “blend in” with the majority, all 20 residents felt they were “acting” or “putting up a façade,” not being their true selves, in fear of judgment and retaliation. Half of the URM residents interviewed felt they had to “act” or “put up a façade” to fit in, reporting often “losing their sense of self-identity in the process.”

Intersectional fatigue/burnout

Overall, eight URM residents reported experiencing “intersectional fatigue/burnout” and “shift anxiety.” Even on off days, they reported feelings of anxiousness about the upcoming interactions with faculty, residents, and staff. Despite adequate rest, these stressors affected their ability to retain orthopedic knowledge and perform effectively in clinical and surgical settings. Identifying as URM, particularly with gender and ethnicity, further compounded stress. One Muslim female resident shared: “I wear a hijab and I’m Muslim, I am female and I am Iranian; yes, I do feel out of place, and I try not to think about it too much, but it is on my mind all the time how different I am in comparison to everyone else.”

Suggested interventions

Participants also recommended solutions to combat burnout and psychosocial stressors. When residents were the only minority in their program, they often sought support from residents in other programs. One Hispanic orthopedic resident reported feeling more comfortable with family medicine residents, as he could be more “comfortable in his own skin.” Many residents proposed the idea of creating discussion groups or forums between residency programs or different specialties within their institution. It was also suggested that such support could be extended to other URMs nationwide through virtual platforms like Zoom, allowing residents to share experiences and support one another.

Female residents in surgical fields, often subjected to micro-aggressions, suggested monthly inter-specialty meetings, such as a forum for women in surgery, where female residents from various specialties could meet to discuss solutions and offer mutual support. Similarly, another resident suggested URMs could benefit from such forums, either virtually or in-person, to create a sense of solidarity and provide support in addressing challenges unique to their demographic.

Many residents emphasized the importance of fostering relationships and finding common interests with faculty and co-residents in a relaxed environment. Residents who shared common interests with faculty found a more satisfactory workplace and clinical interactions and experienced less “awkwardness and tension.” One resident stated: “Having varied activities or events that match different resident interests allows everyone to bond, interact and understand one another; there’s common ground.”

Finally, all 20 interviewed residents emphasized the importance of diversity at leadership levels in promoting wellness and reducing burnout. Residents from programs with diverse leadership expressed higher satisfaction and better well-being compared to those in more homogenous programs. One resident stated: “When faculty members reflect the diverse identities of residents, it displays to residents that the program is seeking to take action and rather than checking an ACGME box or set a façade.”

## Discussion

This qualitative study and thematic analysis of transcripts from 20 URM orthopedic residents highlights their multifaceted experiences and sheds light on the non-orthopedic psychosocial stressors they face due to their racial, gender, religious, and cultural identities, and how these impact burnout and well-being.

The ever-increasing diversity of orthopedic surgery residency programs offers a unique opportunity to explore how gender, religion, and minority status affect resident wellness and burnout. Our results offer a “behind the program” view into the struggles and vulnerabilities that orthopedic surgery trainees from underrepresented backgrounds experience. Themes such as intersectional fatigue, magnifying glass phenomena, self-fulfilling prophecy, code-switching, stereotype threat, and fear of otherness illustrate the psychological, social, and emotional burdens placed on URM residents, ultimately contributing to burnout and diminished professional satisfaction.

Multiple residents in orthopedic programs reported heightened mental fatigue due to the daily stressors associated with their identities, further exacerbating feelings of isolation [36]. This fatigue significantly impacts their well-being, mental bandwidth, and learning capabilities. Residents shared how constant awareness of bias and discrimination led to a “mental fog,” affecting their ability to retain orthopedic knowledge and making study efforts sometimes feel futile [37]. Many residents who reported intersectional fatigue noted how they were unable to be vulnerable with their co-residents, feeling others couldn’t understand their experience of residency as a URM [37]. Our findings are consistent with these studies and suggest that clinical, surgical, and research obligations, compounded with additional stressors faced by URM residents, contribute to a perpetual cycle of emotional catch-up.

Residents who were dismissed or knew of others dismissed from orthopedic residencies frequently cited the “magnifying glass effect,” wherein mistakes were interpreted as indicative of incompetence, leading to a negative reputation and, in some cases, dismissal [31]. The perception that minor mistakes by URM residents were more significant than those of their peers further perpetuates imposter syndrome and affects clinical performance [37].

Throughout our study, we found that many URM residents frequently engage in code-switching to fit into the dominant culture within residency programs. This adaptation is mentally exhausting and contributes to feelings of inauthenticity and burnout. Some residents who struggled with code-switching found the transition to residency especially difficult. Those who could code-switch more easily adapted to varying cultural norms and were more successful in social and professional settings. Residents unable to code-switch often found the residency transition especially challenging when interacting with patients [34]. However, code-switching as it relates to residents interacting with superiors and colleagues has yet to be studied.

Interestingly, two residents of the same ethnicity and program, but from different upbringings, had contrasting experiences with “code-switching.” One had a diverse background and was able to adapt to varying cultural norms. The other, raised in a homogenous inner-city environment, struggled with code-switching and experienced more isolation and imposter syndrome. Residency requires not only academic proficiency but also navigating the “hidden curriculum,” where residents often feel they must “play the game” to succeed [35].

While code-switching can be beneficial, it also leads to multifaceted implications. Medicine and higher education possess deeply rooted cultural norms, often tailored to a specific demographic majority [37]. As diversity and URM representation increase, these norms clash and are not as fluid when assimilating different backgrounds.

Negative stereotypes and stereotype threat have been linked to decreased work performance, and nearly all URM residents voiced feeling vulnerable to stereotype threat [33]. According to Burgess, stereotype threat is more likely to occur when an individual is the only minority of their group, or they are the “token minority” [33]. In our study, participants felt similarly. Residents who were the only minority in their program often felt that they “carried the expectations of their race.” They believed their success or failure would impact future applicants from their demographic. Conversely, URMs in more diverse programs reported feeling less scrutinized.

Programs where diversity, receptiveness, and inclusion were prioritized showed higher satisfaction among residents [38]. Informal wellness and social events catering to diverse interests (cuisine, sports, hobbies, music, etc.) reportedly helped foster stronger relationships among residents and faculty, leading to reduced anxiety, burnout, and stress. Residents felt more comfortable and less isolated, which allowed them to remain authentic and focus on orthopedics rather than fitting into a mold they assumed others expected. This finding is consistent with the study conducted by Frederico et al., where researchers found that when programs focus on promoting resident camaraderie, investment from program and departmental leadership, and a culture of inclusiveness, understanding, and acceptance, residents’ rates of burnout are likely to decrease [39].

This study has several limitations that should be considered when interpreting the findings. First, the sample size was limited to 20 residents from eight orthopedic programs, which may not fully represent the experiences of all URM residents in orthopedic residency programs nationwide. Therefore, the findings may not be generalizable to all residency programs or specialties.

Second, the study relied on self-reported data collected through Zoom interviews, which could subject interviewees to response bias. This self-reported data may also be influenced by the interviewer and the power dynamic that may be created. Participants may have presented their experiences in a way that aligns with social expectations or withheld details due to concerns about confidentiality, stigma, or potential repercussions. The emotional toll of discussing sensitive topics such as discrimination, isolation, and burnout may have also influenced the openness of responses. The power dynamic between the interviewer and the subjects was limited, however, as the position of the interviewer was not revealed during the interview.

The semi-structured interview format allowed for in-depth exploration of individual experiences, but it may have limited the scope of responses. Certain issues or perspectives may not have been fully explored, and participants’ experiences may have been influenced by their specific residency programs or geographic locations, limiting the broader applicability of the findings.

Lastly, the study did not account for potential differences in how intersectional fatigue, stereotype threat, or other factors may manifest across different stages of residency, nor did it explore long-term impacts beyond residency training. The cross-sectional nature of the study captures a snapshot of participants’ experiences during a specific period but does not provide a comprehensive understanding of how these challenges may evolve over time or affect long-term career development.

These limitations suggest the need for further research with a larger, more diverse sample and longitudinal designs to better understand the persistent barriers and coping strategies of URM residents in orthopedic surgery and other medical fields. However, by gaining insight into the direct perspective of orthopedic surgery residents from eight separate institutions, we believe our study contributes valuable insights to the experiences of URM orthopedic residents and provides suggestions that can help address burnout and attrition in orthopedic residencies.

## Conclusions

All interviewed residents emphasized the importance of diversity in leadership as a solution to alleviating URM burnout and isolation. Residents in programs with diverse leadership felt supported and “had someone in their corner,” which propelled them to succeed. Addressing the non-academic challenges faced by URM residents can help foster a more equitable environment that promotes mental health, professional growth, and well-being. Static factors like program location exist, and dynamic elements intrinsic to program culture can be modified to foster a more inclusive environment. However, according to those interviewed, interventions at the leadership level to reduce burnout and psychosocial stress may help mitigate burnout and support retention rates, ultimately promoting diversity retention and allowing residents to thrive. By fostering such an environment, residency programs can produce competent, compassionate, and skilled orthopedic surgeons.

## Appendices

Interview questions

**Table 4:** Interview questions. Credits: Michael Hall Jr., M.D.

Interview questions
What phase/year are you in your training?
What ethnicity or backgrounds/beliefs do you identify yourself with?
Do you consider yourself “religious,” or do you associate yourself with any religious affiliation or denomination?
Does your residency program have a religious affiliation?
Did religious/spiritual beliefs influence your choice to attend LLU? If so, please elaborate.
What are the religious beliefs of your institution? Do you discuss them with attendings and other co-residents? Please elaborate.
If so, how has this religious affiliation affected your residency and interactions with other attendings and residents?
Any positive or negative experiences because of this? If so, please elaborate.
Do you feel isolated from your co-residents due to your religious/spiritual beliefs? OR do you feel they contribute to “closeness” you feel with other co-residents? If so, please explain
How have your religious beliefs/values impacted your residency experience or interactions with patients, attendings, co-residents? Any positive or negative experiences? If so, explain.
Do you feel one spiritual beliefs and “lifestyle” contribute to one’s wellness and ability to cope with residency trial and tribulations? In what ways has it helped you?
Do you know any other residents who are committed to their spiritual beliefs? How so, what is the “majority” in your residency?
What is the demographic/gender makeup of your program? Backgrounds? Religious Affiliations that you may know of?
Do you feel your URM status, gender or religious beliefs have affected your ability to integrate within your program and “fit in”? If so, how?
How does your program seek to include wellness into the curriculum?
At any point during residency have you experienced burnout? If so, how have you been able to cope with it?
At any point in residency have you felt depressed? If so, what strategies have you utilized to help cope?
When it comes to depression, isolation or burnout, how has your program enacted strategies towards helping with such matters? How can it improve?
Why do you think residents who are female or from minority backgrounds/beliefs are more likely to experience burnout, depression, and isolation?
What are some events that the program holds for wellness?
What is the overall comradery of the residents and attendings at your program and what are some ways your program can improve resident camaraderie and wellness?
Do you feel these events are representative of everyone’s common interests?
Have you ever felt isolated during residency? If so, why? If not, what do you feel has helped you not experience feelings of isolation?
Have you ever felt the feelings of imposter syndrome, if so why? What coping mechanisms have you utilized?
What do you think can help negate feelings of imposter syndrome?
Do you have children or an immediate family to care for while in residency? If so, how has this affected your overall well-being in residency?
Do you sometimes feel isolated due to family obligations? If so, elaborate.
Do you feel your residency is understanding of such personal matters or life events that have happened while in residency, if so explain? If not, explain as well.
What is your perception of your program when it comes to diversity inclusion? Do you feel supported?
If you could be program director or chairman and change any aspect of your program, what would it be and why?
Do you feel your expectations about the program were not met when you arrived, in other words, does your program’s current reality meet the expectations/vision that you held while interviewing?
If you had the choice, would you transfer to another program or choose another program if you could?
Do you feel you can comfortably talk to co-residents or faculty about any issues/problems that may arise? Any examples?
Why do you think attrition rates are higher for female orthopedic residents and URM orthopedic residents? What do you think is the solution?
At any point during your residency, did you ever feel that you were considered for dismissal from the program, or did you ever personally consider resigning from the program, if so, why?
Do you know anyone that has been dismissed from their residency program, if so, does it and how does that affect you?
Did you ever feel any one of the faculty members discriminated against, targeted or treated you unfairly? Do you feel it was justified?
Any last remarks or comments from any of the questions/responses?

I
